# Myofilament-level effects of aficamten increase diastolic chamber volumes and maintain cardiac output through preserved length-dependent force generation in healthy rat and canine myocardium

**DOI:** 10.1007/s00395-026-01194-5

**Published:** 2026-06-26

**Authors:** Fruzsina Sárkány, Dániel Priksz, Béla Juhász, László Ádám Fazekas, Norbert Németh, Norbert Szentandrássy, Balázs Horváth, Zsigmond Máté Kovács, Attila Borbély, Arnold Péter Ráduly, Attila Tóth, Miklós Fagyas, Beáta Bódi, Zoltán Papp

**Affiliations:** 1 Division of Clinical Physiology, Department of Cardiology, Faculty of Medicine, University of Debrecen, Móricz Zs. Krt. 22, Debrecen, 4032 Hungary; 2 Kálmán Laki Doctoral School of Biomedical and Clinical Sciences, University of Debrecen, Debrecen, 4032 Hungary; 3 Department of Pharmacology and Pharmacotherapy, Faculty of Medicine, University of Debrecen, Debrecen, 4032 Hungary; 4 Department of Operative Techniques and Surgical Research, Faculty of Medicine, University of Debrecen, Debrecen, 4032 Hungary; 5 Doctoral School of Clinical Medicine, University of Debrecen, Debrecen, 4032 Hungary; 6 Department of Basic Medical Sciences, Faculty of Dentistry, University of Debrecen, Debrecen, 4032 Hungary; 7 Department of Physiology, Faculty of Medicine, University of Debrecen, Debrecen, 4032 Hungary; 8 Division of Cardiology, Department of Cardiology, Faculty of Medicine, University of Debrecen, Debrecen, Hungary

**Keywords:** Myosin inhibitors, Aficamten, Cardiac contractility, Negative inotropy, Length-dependent activation

## Abstract

Clinical trial experience supports the use of myosin inhibitors in hypertrophic cardiomyopathy (HCM), while early proof-of-concept studies suggest potential benefit in selected patients with heart failure with preserved ejection fraction (HFpEF). However, concerns about systolic dysfunction arising from their negative inotropic effects may limit broader application. In a series of in vivo and in vitro experiments in healthy animal preparations, we investigated how aficamten, a next-generation cardiac myosin inhibitor, interacts with key determinants of myocardial contractile function. Echocardiographic examinations in sedated Wistar rats revealed that bolus administration of aficamten (2 mg·kg^−1^ i.v.) did not reduce cardiac output (CO) at pacing frequencies of 300 and 400 bpm, despite marked increases in end-diastolic (ED) and end-systolic left ventricular (LV) volumes and the associated decrease in LV ejection fraction (EF). In field-stimulated intact cardiomyocytes from Mongrel dogs, increasing aficamten concentrations (0.1–1 µM) decreased contraction amplitude and diastolic duration. These effects occurred across pacing frequencies (0.25–1.25 Hz) and in the absence of aficamten-specific changes in intracellular Ca^2+^ transients. In permeabilized LV cardiomyocytes from dog and rat hearts, aficamten decreased active force production (F_max_) and its Ca^2+^ sensitivity under isometric conditions in a similar manner. Importantly, the sarcomere length dependence of F_max_ was preserved, while the length-dependent increase in Ca^2+^ sensitivity was attenuated at higher (0.1–0.2 µM) aficamten concentrations in both species. Our findings in healthy myocardium characterize aficamten as a myosin inhibitor that increases diastolic chamber volumes through reduced contractility, with diastolic function indices (i.e. E/e', IVRT) reflecting the expected hemodynamic consequences of increased preload rather than direct impairment of myofibrillar relaxation. This enhanced preload recruits preserved sarcomere length-dependent force generation to compensate for negative inotropy, thereby maintaining stroke volume and cardiac output.

**Supplementary Information:**

The online version contains supplementary material available at https://doi.org/10.1007/s00395-026-01194-5.

## Introduction

Selective cardiac myosin inhibition has recently emerged as a promising therapeutic concept aimed at reducing excessive cross-bridge formation and normalizing sarcomere function [10, 23]. Growing experience with mavacamten—the first FDA-approved cardiac myosin inhibitor (CMI)—now strongly supports its clinical applicability [30, 32]. In healthy animal models, aficamten, a next-generation CMI, has also demonstrated favorable effects in preclinical and early clinical studies by reducing hypercontractility, relieving left ventricular outflow tract obstruction (LVOTO), and improving exercise capacity in patients with obstructive HCM (oHCM) [1, 7, 30, 36, 37]. Because heart failure with preserved ejection fraction (HFpEF) often involves impaired diastolic relaxation and increased ventricular stiffness—pathophysiological features that in some patients may resemble the diastolic abnormalities in HCM—CMIs have been also proposed as a potential therapy to improve myocardial relaxation and reduce wall stress in HFpEF [10, 12]. Indeed, the recent phase 2a proof-of-concept EMBARK-HFpEF trial showed that 26 weeks of treatment with mavacamten in patients with symptomatic HFpEF (LVEF ≥ 60%) was associated with reductions in circulating levels of N-terminal pro-brain natriuretic peptide (NT-proBNP) and troponin and modest improvements in diastolic function and New York Heart Association (NYHA) class, without a sustained drop in LVEF [43]. Although promising, these results remain preliminary, and broader applicability of CMIs in HFpEF will require further randomized trials and careful patient selection [19].

An important unresolved general concern is whether myosin-targeted therapy may precipitate hemodynamic deterioration due to its negative inotropic action. This concern is underscored by the prescribing recommendations for mavacamten. Treatment initiation is contraindicated when LVEF is < 55%, and therapy must be interrupted if LVEF decreases to < 50% during follow-up [14, 17]. These restrictions were derived from clinical trial experience and adverse event monitoring. Yet it remains uncertain whether LVEF alone is an adequate or physiologically grounded parameter for guiding the safe use of CMIs, given that systolic performance depends on multiple interacting determinants.

Cardiac output and contractile reserve are modulated not only by myofilament activation but also by neurohormonal regulation (primarily via the β-adrenergic system) and the force-frequency relationship (FFR) through effects on Ca^2+^ homeostasis [9, 26]. In addition, mechano-functional feedback mechanisms such as the Anrep effect and the Frank-Starling mechanism adjust myocardial performance in response to acute changes in loading conditions [39]. How these intrinsic regulatory pathways integrate with pharmacological myosin inhibition, and whether they can compensate for the drug-induced reduction in cross-bridge cycling, remains largely unexplored. Importantly, such compensatory mechanisms may determine whether negative inotropy translates into reduced cardiac output or is offset by enhanced ventricular filling and preserved stroke volume.

In the present study, we investigated the interaction between aficamten-induced myosin inhibition and the major regulators of myocardial contractile function using a set of complementary cardiac preparations spanning different levels of physiological complexity in rat and canine hearts. By applying a range of aficamten concentrations and pacing conditions, we aimed to identify the key determinants of its effects on systolic and diastolic performance and to define how myosin inhibition integrates into whole-organ hemodynamics. Our findings provide mechanistic insight into how aficamten may preserve cardiac output despite its inhibitory action on myosin, thereby informing the physiological basis for safe and effective therapeutic application.

## Materials and methods

### Animals

In vivo experiments and left ventricular (LV) myocardial tissue collection for permeabilized cardiomyocyte studies were performed in adult male Wistar rats (10–12 weeks old; n = 20; body weight 489.23 ± 27.99 g; Toxi-Coop Zrt., Budapest, Hungary). Animals were housed in the institutional animal facility under controlled ambient temperature and a 12/12 h light–dark cycle, with free access to standard chow and water. Intact ventricular cardiomyocytes for cell shortening and intracellular Ca^2+^ measurements were isolated from adult mongrel dogs of either sex (n = 6 dogs; body weight 10.0 ± 3.0 kg; age 15 ± 1 months; obtained from a local breeder). All procedures conformed in accordance with national (Act XXVIII of 1998 on the Protection and Sparing of Animals) and EU (Directive 2010/63/EU) regulations for the care and use of laboratory animals and were approved by the local Animal Care Committee and by the National Food Chain Safety Office (registration Nr. 2/2020/UDCAW; 1/2024/UDCAW).

### Transthoracic echocardiography during intracardiac pacing

Transthoracic echocardiography was performed in rats anaesthetized with a ketamine/xylazine mixture (100/10 mg·kg⁻^1^, i.p., CP ketamine hydrochloride 10%/CP xylazine hydrochloride, 2%; Produlab Pharma BV, Raamsdonksveer, Amsterdam, the Netherlands) [35]. After the chest hair was removed and the tail vein was cannulated (26 G, Neoflon^TM^ Pro IV Cannula; Becton, Dickinson and Company, Franklin Lakes, NJ, USA) animals were positioned supine on a temperature-controlled imaging platform (SR200, VisualSonics, Amsterdam, the Netherlands) maintained at 39 °C. A three-lead surface ECG was continuously recorded throughout the procedure.

A temporary single-chamber external cardiac pacemaker (Medtronic, Galway, Ireland) was used for electrical stimulation (240, 300, 400 Hz) at the minimum effective voltage (~ 4 V). The ventral cervical region, extending from the sternum to the mandible, was prepared using an electric trimmer (MG3920/15, Philips, Amsterdam, the Netherlands) and depilatory cream (Veet, Reckitt Benckiser, Tatabánya, Hungary). Following cutaneous disinfection with povidone-iodine, an incision was performed to expose the right external jugular vein, which was subsequently dissected from the surrounding tissue. The vessel was ligated (7–0 Pidilen, Kollsut USA, Miami, USA) just below the junction with the internal jugular vein, and a bipolar pacing catheter (52124N9, Bipolar EndoStim^TM^, OSYPKA Medical, Berlin, Germany) was introduced. The placement of the pacing electrode within the right atrium was validated in each instance via echocardiography and electrocardiogram (ECG) monitoring, after which the catheter was anchored with two ligatures.

Echocardiographic imaging was carried out using the Vevo 3100 Imaging System and Vevo Imaging Station (VisualSonics, Amsterdam, the Netherlands) equipped with a high-frequency MX250 transducer (14–28 MHz). Imaging initially involved determination of drug-free control parameters and was subsequently initiated 5 min after intravenous administration of aficamten (2 mg·kg⁻^1^) [6, 51], with the entire protocol lasting no longer than 15 min. Data were acquired in B-mode and M-mode, from parasternal long- and short-axis (PSLAX and PSAX) views. Cardiac function was assessed in accordance with the recommendations of the American Society of Echocardiography [31]. Manufacturer-recommended settings for focus, 2D gain, image width, and depth were applied to optimize B-mode quality. Heart rate (beats per minute, bpm) was automatically derived from ECG R–R intervals averaged over a 5 s segment. LV wall thickness and chamber dimensions were measured in M-mode at the mid-papillary level from both PSLAX and PSAX views. End-diastolic and end-systolic diameters (EDD and ESD, mm) were obtained by manual tracing of endocardial and epicardial borders on PSAX M-mode images. End-diastolic and end-systolic volumes (EDV and ESV, µL) were calculated from PSLAX delineations. Stroke volume (SV, µL) was determined as SV = EDV − ESV. Cardiac output (CO, mL/min) was estimated as SV × HR / 1000. LV fractional shortening (FS, %) was determined as FS = 100 × (EDD − ESD)/EDD, and LV ejection fraction (EF, %) was determined as EF = 100 × (EDV − ESV)/EDV. Global longitudinal strain (GLS, %) was quantified using the strain analysis module (Vevo Strain) of the Vevo LAB 5.6.0 software (VisualSonics, Amsterdam, the Netherlands) by manually delineating endocardial contours. GLS was determined by the software as follows: GLS = (L_ES_ – L_ED_)/L_ED_, where L_ED_ represents the original length of the LV contour (at the R-wave in diastole) and L_ES_ represents the length of the LV contour in systole. For each parameter, three consecutive cardiac cycles were averaged. Left ventricular diastolic function was assessed via Doppler echocardiography. From the apical four-chamber view, pulsed-wave Doppler was used to determine peak early diastolic mitral inflow velocity (E), while tissue Doppler imaging (TDI) was applied to measure peak early diastolic mitral annular velocity (e') at the septal annulus; the E/e' ratio was subsequently calculated as an index of left ventricular filling pressure. TDI at the septal annulus was used to determine isovolumetric relaxation time (IVRT), isovolumetric contraction time (IVCT), and ejection time (ET). Systolic duration was calculated as the sum of ET and IVCT (ET + IVCT), while diastolic duration was defined as the total cardiac cycle length minus the systolic duration.

### Isolation of canine left ventricular cardiomyocytes with intact membranes

Hearts were excised from adult mongrel dogs anaesthetized with intramuscular ketamine hydrochloride (10 mg·﻿kg⁻^1^; CP-Ketamin, Produlab Pharma BV, Raamsdonksveer, the Netherlands) and xylazine hydrochloride (1 mg·kg⁻^1^; CP-Xylazin, Produlab Pharma BV, Raamsdonksveer, the Netherlands). Ventricular cardiomyocytes were isolated from the midmyocardial region of the left ventricle as described previously [13, 18]. Briefly, the left anterior descending coronary artery was cannulated, and the tissue was initially perfused with Ca^2+^-free Joklik solution (Minimum Essential Medium Eagle, Joklik Modification; Sigma-Aldrich, St. Louis, MO, USA) for 5 min. This was followed by a 30 min perfusion with a modified Joklik solution containing collagenase (Type II, 1 mg·mL⁻^1^; Worthington Biochemical Co., Lakewood, NJ, USA), 0.2% bovine serum albumin (Fraction V; Sigma), and 50 µM CaCl₂. After enzymatic digestion, the tissue was gently dispersed to obtain single cardiomyocytes. Extracellular Ca^2+^ concentration was then gradually increased in Tyrode solution (containing 144 mM NaCl, 5.6 mM KCl, 2.5 mM CaCl₂, 1.2 mM MgCl₂, 5 mM HEPES, and 11 mM dextrose; pH 7.4; all reagents from Sigma-Aldrich, St. Louis, MO, USA). Isolated cardiomyocytes were stored at 15 °C until use.

### Cell shortening and intracellular Ca^2+^ measurements in intact LV canine cardiomyocytes

Intact LV cardiomyocytes were loaded with the Ca^2+^-sensitive ratiometric fluorescent dye Fura-2 AM (5 µM) in the presence of Pluronic F-127 (25 µg mL⁻^1^) in Tyrode solution to prevent premature dye extrusion. Pluronic F-127 (25 mg) was dissolved in 1 mL DMSO, which was used as the solvent to prepare the Fura-2 AM stock solution. Cells were incubated for 30 min to allow intracellular esterases to de-esterify the dye. After loading, cardiomyocytes were transferred to an experimental chamber mounted on an inverted microscope (Nikon TS-100) and superfused with 1 mL Tyrode solution. Once settled, a rod-shaped cardiomyocyte with clear striations was selected for analysis. Field stimulation was applied at 0.25, 0.5, 0.75, 1.0, and 1.25 Hz (Experimenta Setup, MDE, Heidelberg). Alternating excitation wavelengths of 340 and 380 nm were used to elicit fluorescence from Ca^2+^-bound and Ca^2+^-free Fura-2, respectively. Emission > 510 nm was collected, and optical signals were digitized at 120 Hz using FeliX Software (Ratiomaster RM-50, Horiba, New Brunswick, NJ, USA). At the start of each experiment, cardiomyocytes were stimulated at 0.5 Hz for 2–3 min to achieve steady state. Aficamten was then applied cumulatively to reach final concentrations of 0.1, 0.25, 0.75, and 1 µm, with a 4–7 min incubation after each addition. Baseline fluorescence was measured at the end of each experiment in a cell-free region and manually subtracted from the recorded fluorescence signals for background correction. Experiments were performed at room temperature (22 °C).

Sarcomere length (SL, µm) was measured using a high-speed camera. Contraction duration was defined as the interval from the onset to the end of shortening, whereas time-to-peak shortening was measured from the onset to the peak of contraction. Contraction and relaxation velocities were determined from the linear portions of the shortening and relengthening phases and expressed in µm·s⁻^1^. Resting Ca^2+^ levels were estimated from the baseline Fura-2 fluorescence ratio (340/380). Ca^2+^ transient duration was defined as the time between the 50% points on the upstroke and downstroke of the transient. Transient amplitude was calculated as the difference between peak and baseline Fura-2 ratios (340/380).

### Force measurements in permeabilized rat and canine left ventricular cardiomyocytes

Deep-frozen LV myocardial samples were thawed and homogenized in isolating solution (ISO) containing 1 mM MgCl₂, 100 mM KCl, 2 mM EGTA, 4 mM ATP, and 10 mM imidazole (pH 7.0), supplemented with protease inhibitors (0.5 mM phenylmethylsulfonyl fluoride, 40 µM leupeptin, and 10 µM E-64; all reagents from Sigma-Aldrich, St. Louis, MO, USA). Permeabilization was achieved by incubating the tissue in 0.5% Triton X-100 (Thermo Fisher Scientific, Waltham, MA, USA) for 5 min. Single permeabilized cardiomyocytes were attached using silicone adhesive (DAP 100% all-purpose sealant; Baltimore, MD, USA) between two stainless steel insect needles connected to a high-sensitivity force transducer (SensoNor, Horten, Norway) and an electromagnetic length controller (Aurora Scientific Inc., Aurora, Canada). Isometric force measurements were performed at 15 °C with sarcomere length preset to either 2.3 µm or 1.9 µm. Measurement solutions contained 7 mM CaEGTA for activating conditions (pCa 4.75) or 7 mM EGTA for relaxing conditions (pCa 9.0), 37.34 mM KCl, 10 mM N,N-bis(2-hydroxyethyl)-2-aminoethanesulfonic acid (BES), 6.24 mM MgCl₂, 6.99 mM Na₂ATP, and 15 mM Na₂CrP, adjusted to pH 7.2. Contractile activation was initiated by transferring a cardiomyocyte from relaxing solution (pCa 9.0; pCa = –log[Ca^2+^]) to activating solution (pCa 4.75). Once steady-state force developed, a rapid release–restretch maneuver (30 ms) was applied to determine baseline force, allowing calculation of Ca^2+^-activated total force (F_total_). Approximately 6 s after force redevelopment, passive force (F_passive_) was measured by shortening the preparation to 80% of its initial length for 8 s in relaxing solution (pCa 9.0). Active force (F_active_) was computed as F_total_ – F_passive_. Maximal Ca^2+^-activated force (F_max_) was obtained at pCa 4.75. Submaximal activations (pCa 5.2–7.0) were used to construct the pCa–force relationship, with F_active_ values normalized to F_max_. Relative force values were fitted using a modified Hill equation (Origin 6.0, Microcal Software, Northampton, MA, USA) to determine Ca^2+^ sensitivity (pCa₅₀). Absolute force values were normalized to the cross-sectional area of each cardiomyocyte, calculated from measured width and height, and expressed in kN·m⁻^2^.

### Drugs and chemicals

All chemicals were obtained from Sigma-Aldrich (St. Louis, MO, USA). Aficamten was purchased from MedChemExpress (Monmouth Junction, NJ, USA). For intracellular Ca^2+^ transient and cardiomyocyte shortening measurements, appropriate volumes of concentrated aficamten stock solutions (prepared in DMSO) were diluted in the bathing medium to yield final aficamten concentrations of 0.1, 0.25, 0.4, 0.75, and 1 µM. The final DMSO concentration was 0.1%; vehicle controls contained the same DMSO concentration without aficamten. For isometric force measurements in permeabilized cardiomyocytes, defined amounts of aficamten stock solutions were added to relaxing and activating solutions to obtain final concentrations of 0.001, 0.003, 0.01, 0.03, 0.1, 0.3, 1, 3, and 10 µM. To generate concentration–response curves and estimate the IC₅₀ values of aficamten in rat and canine permeabilized cardiomyocyte preparations, aficamten stock solutions prepared in activating solution (pCa 4.75) were serially diluted and applied to the preparations, followed by determination of maximal force (F_max_).

### Data analysis and statistics

Transthoracic echocardiography was performed in twenty rats before and after intravenous administration of aficamten. In vitro experiments on intact canine cardiomyocytes were conducted using 10–12 cells isolated from six to twelve different hearts (n = 6–12 independent biological replicates). For each experimental condition values were averaged within each heart before statistical comparison if required. Thus, statistical analyses were performed using heart-level averages, treating hearts rather than individual cells as the unit of replication to avoid pseudoreplication. Group data are presented as mean ± SEM from n = 6–12 hearts. Background fluorescence was measured at the end of each experiment in a cell-free region and manually subtracted from the recorded fluorescence signals for background correction. For force measurements in permeabilized rat and canine cardiomyocytes, 6–8 cells originating from three hearts per species were analyzed. Group data are presented as mean ± SEM from n = 3 hearts. Data processing and graphical representations were performed using GraphPad Prism 10.2.3 (GraphPad Software, San Diego, CA, USA). Normality of distributions was assessed using the Kolmogorov–Smirnov test. Depending on data characteristics, comparisons were performed using mixed-effects analysis, two-way ANOVA followed by Tukey’s post hoc test, or the Kruskal–Wallis test with Dunn’s multiple-comparison procedure. Wilcoxon or paired *t*-tests were used to assess aficamten-induced changes within individual animals. A value of P < 0.05 was considered statistically significant.

## Results

### Aficamten increases LV dimensions and reduces LVEF in vivo

**Fig. 1:**
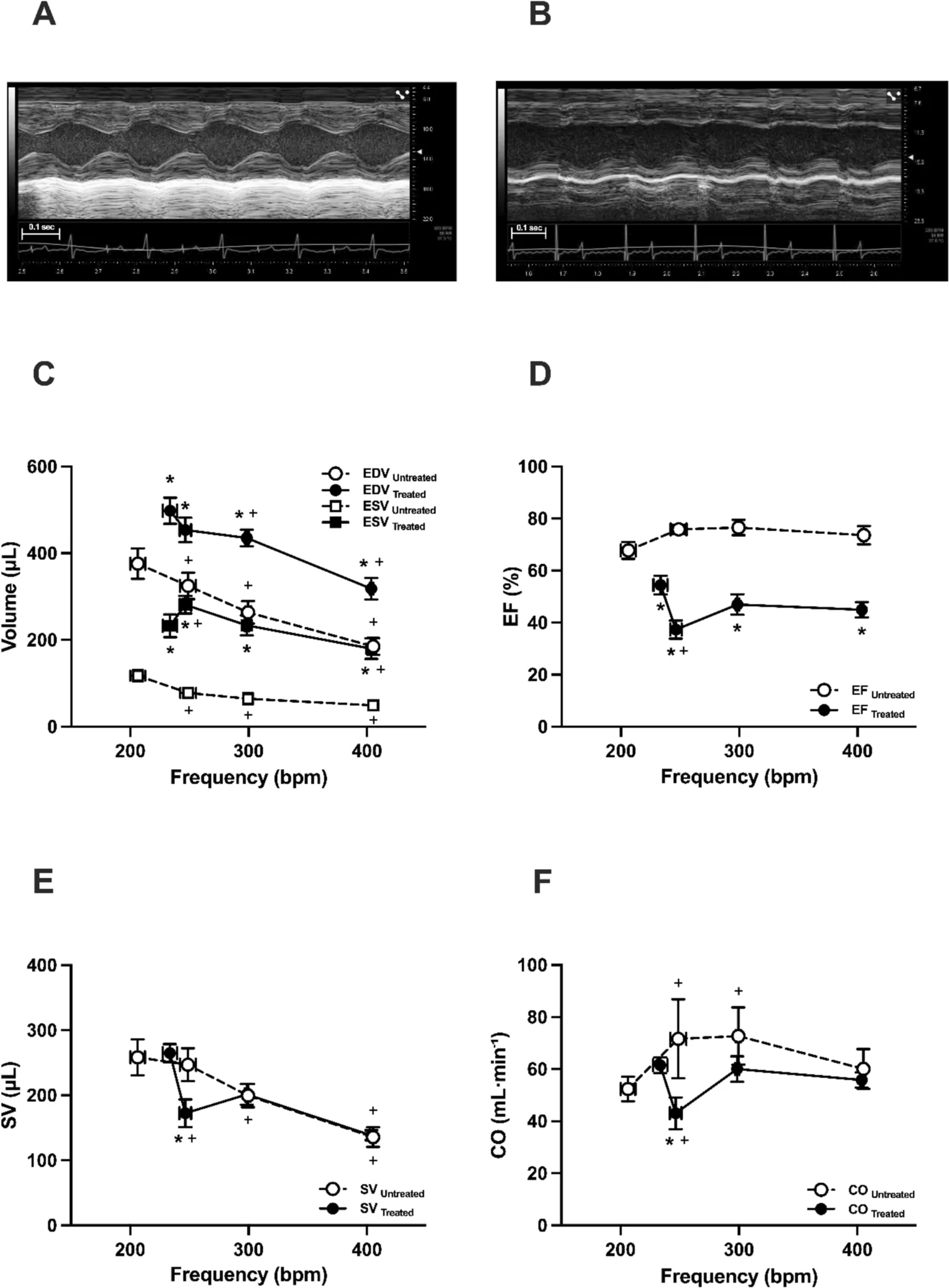
Echocardiographic characterization of aficamten effects on cardiac function in anesthetized rats. Representative M-mode images acquired at 300 bpm illustrate the increases in LV end-diastolic volume (EDV) and end-systolic volume (ESV) observed before (**A**) and after (**B**) aficamten administration (2 mg kg⁻^1^ i.v.) in the same animal. (**C**) Across animals, EDV and ESV showed frequency-dependent declines from baseline heart rate through 400 bpm pacing, both before (open symbols) and after (filled symbols) aficamten treatment. Aficamten increased baseline EDV and ESV while reducing ejection fraction (EF) (**D**) at all heart rates. Despite marked reductions in EF, stroke volume (SV) (**E**) and cardiac output (CO) (**F**) were preserved at 300 and 400 bpm following aficamten administration. Data are presented as means ± SEM from paired within-animal comparisons (n = 10 rats). Lines connect baseline and aficamten values from the same group of animals. Aficamten increased resting heart rate from 206.3 ± 6.3 to 233.6 ± 6.0 bpm, precluding reliable pacing at 240 bpm; therefore, data at 300 and 400 bpm provide the most robust assessment of hemodynamic compensation. *P < 0.05 vs. before treatment. + P < 0.05 vs. baseline heart rate within each condition

The acute effects of aficamten (2 mg·kg⁻^1^ i.v.) on cardiac function were assessed by transthoracic echocardiography in anaesthetized rats at intrinsic heart rate and during intracardiac pacing at 240, 300, and 400 bpm. Aficamten elevated baseline heart rate from 206.3 ± 6.3 bpm to 233.6 ± 6.0 bpm, which resulted in technical challenges for pacing at 240 bpm. At this pacing frequency (only marginally above the drug-induced baseline), intermittent capture failures occurred in 70% of animals (capture success 88–90%), while stable 1:1 capture was achieved at 300 and 400 bpm in all animals (intermittent capture failures in only 20% of animals; success rate 95–100%). Therefore, cardiac output data at 300 and 400 bpm provide the most reliable assessment of aficamten's hemodynamic effects. Despite this technical limitation, prominent LV dilation was consistently observed following aficamten administration at all heart rates (P < 0.05), as illustrated by representative M-mode recordings at 300 bpm (Fig. 1A, B). Both LV end-diastolic volume (EDV) and end-systolic volume (ESV) declined with increasing heart rate under drug-free conditions and after aficamten treatment. Before treatment, EDV decreased from a 376 ± 35 µL baseline to 185.2 ± 19.5 µL at 400 bpm (P < 0.05). Aficamten increased baseline EDV to 498 ± 30.2 µL, which then declined to 318.4 ± 25.1 µL at 400 bpm (P < 0.05) in the same animals. Similarly, ESV declined from 117.7 ± 13.2 µL to 49.3 ± 9 µL before treatment (P < 0.05), and aficamten increased baseline ESV to 233 ± 26.4 µL, declining to 179.8 ± 23.8 µL at 400 bpm (P < 0.05) (Fig. 1C). Under drug-free conditions, EF was 67.8 ± 3.2% at baseline and 73.6 ± 3.52% at 400 bpm (P > 0.05). In contrast, aficamten reduced EF substantially (P < 0.05 vs. control) at all heart rates (54.4 ± 3.6% at baseline and 45 ± 3% at peak heart rate; Fig. 1D). SV declined with increasing heart rate similarly in both groups: from 258.4 ± 27.8 µL to 135.8 ± 15.1 µL before aficamten treatment and from 265 ± 14 µL to 138.5 ± 7.7 µL after aficamten administration (P < 0.05 vs. baseline; Fig. 1E). Changes in cardiac output (CO) were also largely similar (63.8 ± 8.3 to 58 ± 7 mL·min⁻^1^ before treatment; 52.36 ± 4.8 to 60.1 ± 7.8 mL·min⁻^1^ after aficamten; Fig. 1F). Notably, aficamten did not reduce SV or CO at 300 or 400 bpm (Fig. 1E, F). Paired individual animal data for EDV, ESV, SV, and CO at 300 bpm confirmed consistent stroke volume and cardiac output preservation across all animals with stable capture (Supplementary Fig. S1). Additionally, vehicle control experiments yielded qualitatively identical results for EDV, ESV, EF, SV, and CO (data not shown). Furthermore, in the vehicle-controlled study, fractional shortening (FS) increased from 43.0 ± 2.6% to 48.6 ± 3.8% in controls, whereas aficamten markedly suppressed FS (18.8 ± 1.5% at baseline to 19.9 ± 2.4% at 400 bpm; P < 0.05 vs. control at all heart rates). Similarly, global longitudinal strain (GLS) remained relatively preserved in vehicle controls (− 28.5 ± 2.5% at baseline to − 29.7 ± 4.5% at peak pacing), while aficamten significantly shifted GLS toward more positive values at all heart rates (− 18.2 ± 2.7% at baseline to − 13.3 ± 1.8% at peak frequency; P < 0.05 vs. controls).

### Aficamten increases E/e' ratio and prolongs isovolumetric relaxation time

**Fig. 2:**
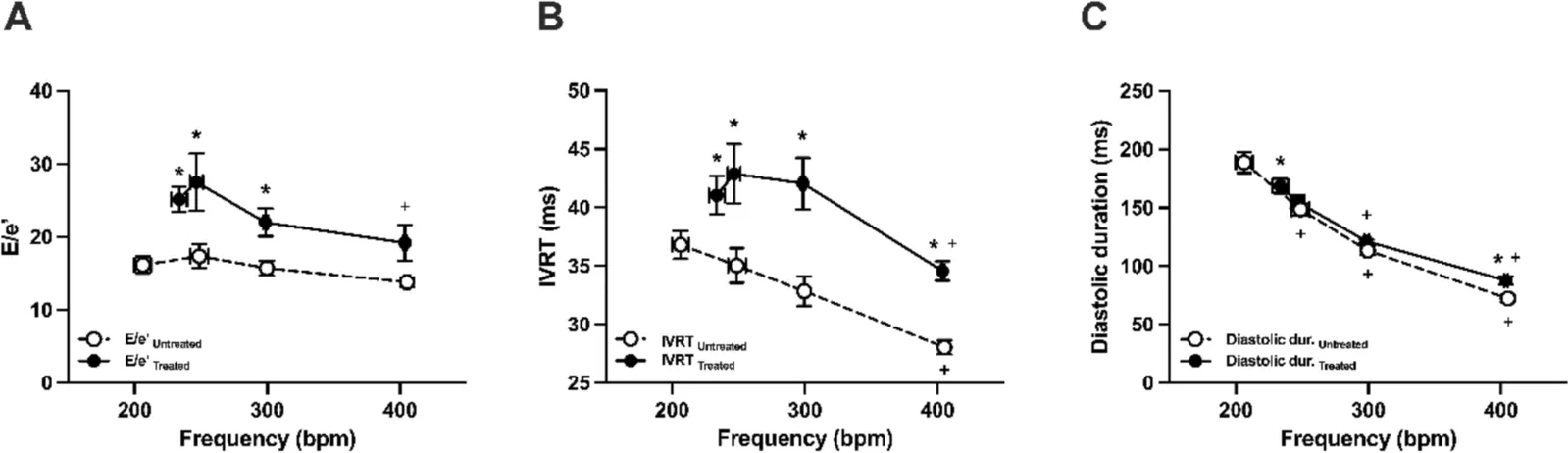
Diastolic filling parameters before and after aficamten. (**A**) E/e' ratio, (**B**) isovolumetric relaxation time (IVRT), and (**C**) diastolic duration in rats before (open symbols) and after (closed symbols) aficamten (2 mg·kg⁻^1^ i.v.) across heart rates. Aficamten increased E/e' and prolonged IVRT at all frequencies without consistently altering diastolic duration. Data are presented as means ± SEM from paired within-animal comparisons (n = 10 rats). Lines connect baseline and aficamten values from the same group of animals. *P < 0.05 vs. before treatment. + P < 0.05 vs. baseline heart rate within each condition

In the absence of aficamten, E/e' remained stable with increasing heart rate (16.2 ± 1.1 at baseline and 13.8 ± 0.7 at 400 bpm), but increased following aficamten administration in the same animals (25.1 ± 1.7 at baseline and 19.2 ± 2.4 at 400 bpm, P < 0.05 vs. drug-free; Fig. 2A). In the absence of aficamten, IVRT decreased with increasing pacing frequency, reaching statistical significance at 400 bpm (36.8 ± 1.2 ms at baseline vs. 28.0 ± 0.6 ms at 400 bpm, P < 0.05). Aficamten treatment prolonged IVRT at all heart rates (41 ± 1.6 ms at baseline and 34.6 ± 0.8 ms at 400 bpm, P < 0.05; Fig. 2B). Diastolic durations declined similarly with increasing pacing frequency in both conditions (drug-free: 188.8 ± 9.1 ms at baseline to 72.4 ± 3.1 ms at 400 bpm; aficamten: 168.8 ± 6.2 ms at baseline to 87.7 ± 3.6 ms at 400 bpm; Fig. 2C), with no consistent effect of aficamten across heart rates. These findings indicate that aficamten increased left ventricular filling pressures (elevated E/e') and prolonged isovolumetric relaxation without extending diastolic filling time, suggesting that the observed increase in EDV results from elevated filling pressures rather than prolonged filling duration.

### Aficamten reduced cardiomyocyte contractility in a concentration-dependent manner without affecting intracellular Ca^2+^ transients

**Fig. 3:**
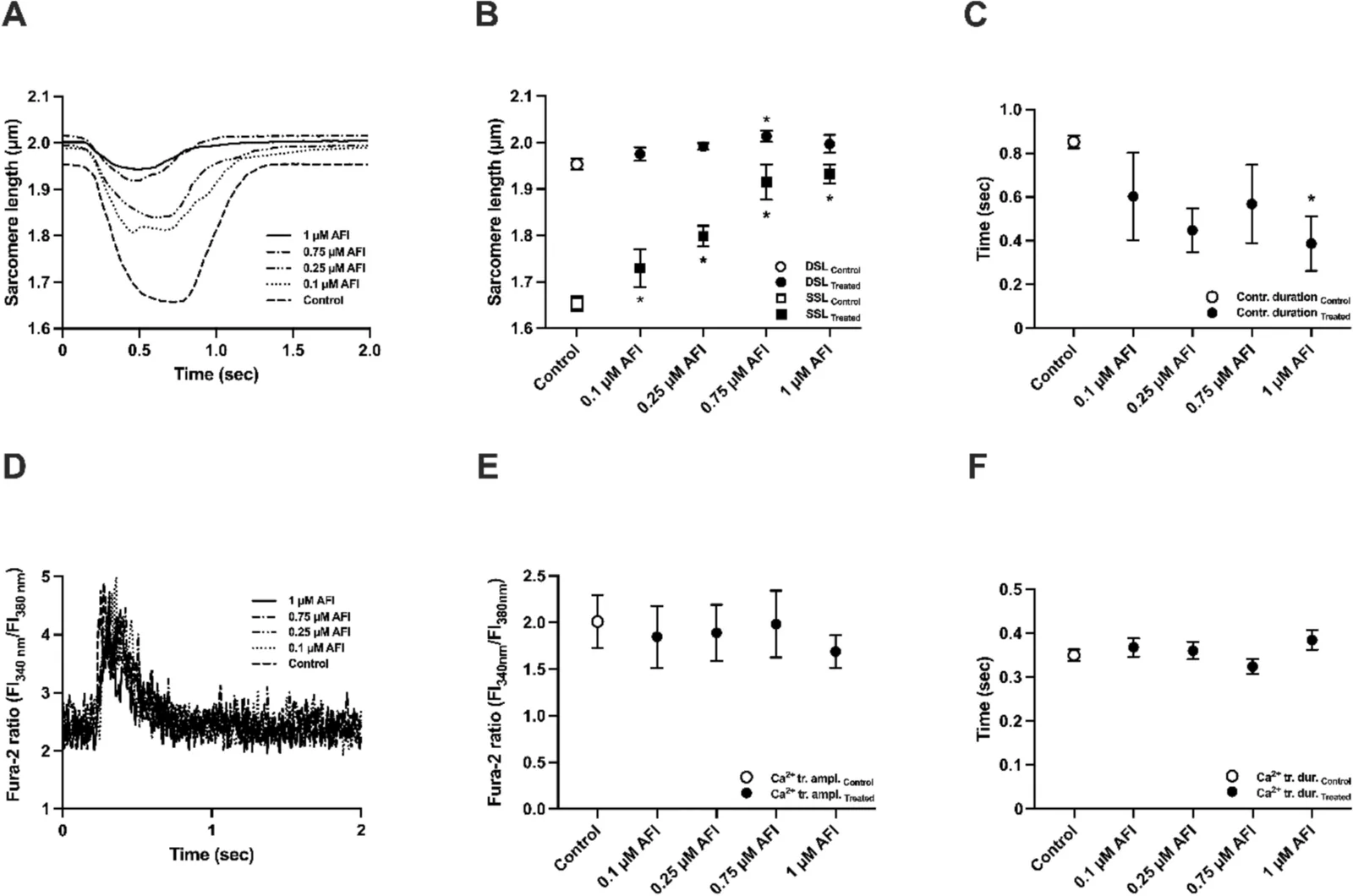
Concentration-dependent effects of aficamten in field-stimulated (0.5 Hz) single left ventricular canine cardiomyocytes with intact membranes. Representative sarcomere length (SL) traces illustrate the progressive effects of increasing aficamten concentrations on cellular shortening (**A**). Aficamten decreased both systolic and diastolic SL (SSL and DSL, respectively) in a concentration-dependent manner (**B**) and reduced contraction duration (**C**). Corresponding intracellular Ca^2+^ transient recordings are shown in (**D**). In contrast to its mechanical effects, aficamten did not alter Ca^2+^ transient amplitude or duration (**E**, **F**). Data are presented as mean ± SEM from n = 6–12 hearts (10–12 cells total per species). Significant differences between aficamten-treated cells (closed symbols) and drug-free controls (open symbols) are indicated by asterisks (*P < 0.05)

The effects of aficamten on contractile function and intracellular Ca^2+^ handling were assessed in isolated intact canine LV cardiomyocytes paced at 0.5 Hz at room temperature. Sarcomere length (SL) and Ca^2+^ transients were recorded under baseline conditions and after administration of 0.1, 0.25, 0.75, and 1 µM aficamten. Representative traces illustrate clear concentration-dependent mechanical effects in the absence of detectable changes in Ca^2+^ cycling (Fig. 3A, D). Aficamten induced a concentration-dependent attenuation of systolic sarcomere shortening, with end-systolic sarcomere length increasing from 1.67 ± 0.01 µm in drug-free conditions to 1.93 ± 0.02 µm at 1 µM, reflecting reduced contractile performance (P < 0.05 vs. control for all concentrations). Basal diastolic sarcomere length (DSL) showed a modest but significant concentration-dependent increase, rising from 1.95 ± 0.01 µm in drug-free controls to 2.01 ± 0.01 µm at 0.75 µM (P < 0.05; Fig. 3B). The duration of contraction was significantly shortened by aficamten at 1 µM (P < 0.05). Specifically, contraction duration decreased from 0.85 ± 0.03 s in drug-free controls to 0.39 ± 0.12 s at 1 µM (Fig. 3C). Importantly, aficamten did not alter intracellular Ca^2+^ transients. Neither the amplitude nor the duration of Ca^2+^ signals changed at any applied concentration (Fig. 3E, F), demonstrating that the observed reduction in contractility occurred independently of Ca^2+^ handling. Moreover, the consistency of Ca^2+^ transient parameters across the cumulative incubation protocol confirms preparation stability throughout the experiment.

### Aficamten preserves frequency-dependent contractility and Ca^2+^ dynamics

**Fig. 4:**
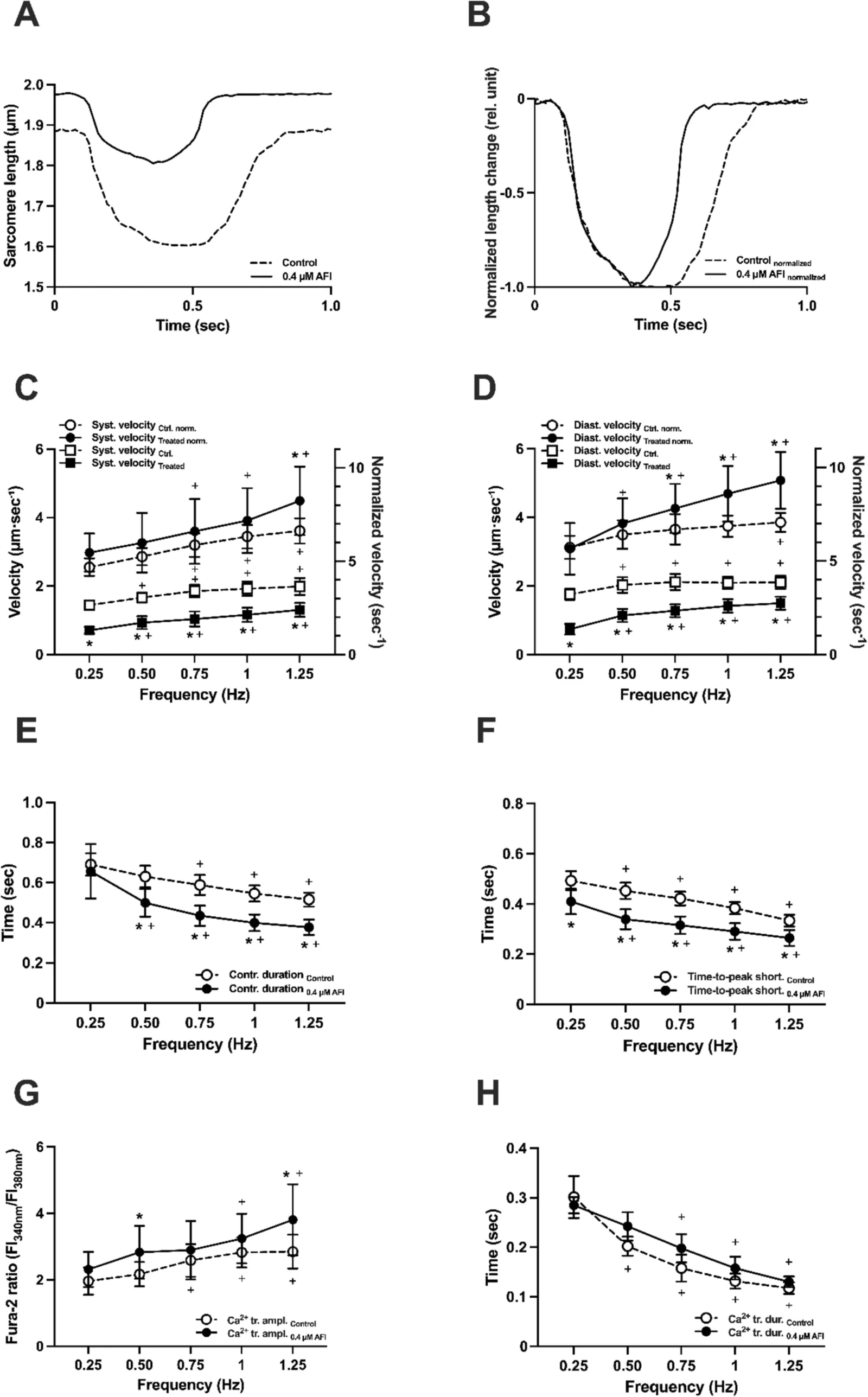
Frequency-dependent effects of aficamten (0.4 µM) in single left ventricular canine cardiomyocytes with intact membranes. Representative original (**A**) and normalized (**B**) sarcomere length (SL) traces demonstrate the effects of aficamten (closed) versus drug-free conditions (open) during steady-state field stimulation at 1 Hz. Squares show absolute contraction (**C**) and relaxation (**D**) velocities, while circles show values normalized to the same shortening amplitude. Aficamten reduced both contraction and relaxation velocities when expressed in absolute terms (**C**, **D**) across all pacing frequencies. After normalization, contraction and relaxation velocities were similar to drug-free conditions (**C**, **D**). Contraction duration (**E**) and time to peak shortening (**F**) decreased progressively with increasing stimulation frequency under control conditions, and aficamten induced additional significant reductions in contraction duration at ≥ 0.5 Hz and in time to peak shortening at ≥ 0.25 Hz. Field stimulation produced a frequency-dependent increase in Ca^2+^ transient amplitude at ≥ 0.75 Hz and a decrease in Ca^2+^ transient duration at ≥ 0.5 Hz in untreated cells. Aficamten did not consistently alter Ca^2+^ transient amplitude across pacing frequencies (**G**), and Ca.^2+^ transient duration was unaffected at all frequencies tested (**H**). Data are presented as mean ± SEM from n = 6 hearts (1–2 cells per species). Significant differences between aficamten-treated and control groups are indicated by asterisks (*P < 0.05). Significant frequency-dependent changes relative to baseline within each group are indicated by crosses (+ P < 0.05)

To assess the interaction between aficamten and cardiac frequency–response dynamics, intact canine LV cardiomyocytes were stimulated between 0.25 and 1.25 Hz in the presence of a single submaximal concentration of the drug (0.4 µM). Representative absolute and normalized SL traces at 1 Hz pacing illustrate clear kinetic differences (Fig. 4A, B). Although aficamten appeared to slow contraction and relaxation in absolute terms, normalization to identical shortening amplitudes revealed that the drug did not impair the intrinsic contraction–relaxation kinetics. Consistent with this, normalized peak contraction velocity increased from 4.68 ± 0.47 s⁻^1^ at the drug-free baseline (0.25 Hz) to 6.62 ± 0.67 s⁻^1^ at 1.25 Hz (P < 0.05). In the presence of aficamten, normalized peak contraction velocity similarly increased from 5.46 ± 1.02 s⁻^1^ to 8.23 ± 1.83 s⁻^1^ across the same frequency range (P < 0.05 vs. baseline; Fig. 4C). Normalized peak relaxation velocity showed a comparable pattern: values increased from 5.73 ± 0.61 s⁻^1^ to 7.06 ± 0.51 s⁻^1^ in drug-free conditions (P < 0.05), and from 5.65 ± 1.38 s⁻^1^ to 9.31 ± 1.52 s⁻^1^ in the presence of aficamten (P < 0.05; Fig. 4D). Application of 0.4 µm aficamten significantly shortened contraction duration across frequencies between 0.5 and 1.25 Hz and time-to-peak between 0.25 and 1 Hz (P < 0.05), and these effects were not constrained by the frequency dependence of either parameter (Fig. 4E, F). Importantly, the frequency-dependent increase in peak intracellular Ca^2+^ transient amplitude was largely preserved in the presence of aficamten. Moreover, no significant differences and the frequency-dependent decrease in Ca^2+^ transient duration were observed between drug-free and aficamten-treated cardiomyocytes at any pacing frequency (P > 0.05; Fig. 4G, H). Supplementary Figs. S2, S3 illustrate the frequency-dependence (0.25–1.25 Hz) of contractile kinetics and Ca^2+^ transient responses, respectively, under control and aficamten conditions, showing consistent drug effects across hearts alongside inter-heart variability.

### Aficamten induces comparable concentration-dependent decreases in F_active_ and Ca^2+^ sensitivity in permeabilized rat and canine cardiomyocytes

**Fig. 5:**
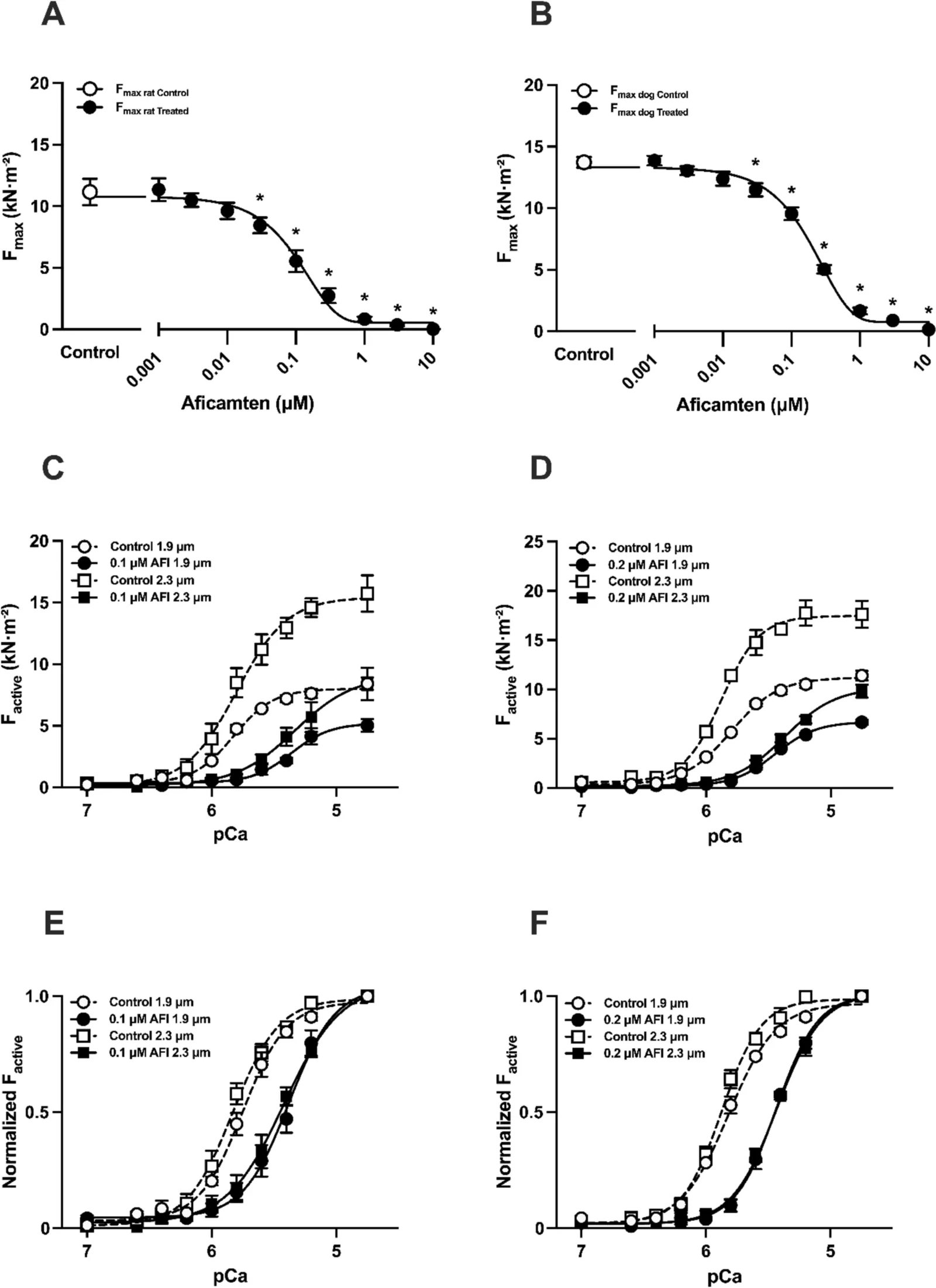
Concentration-dependent effects of aficamten on isometric active force production in permeabilized single left ventricular rat (**A**, **C**, **E**) and canine (**B**, **D**, **F**) cardiomyocytes. Aficamten (0.001–10 µM) reduced maximal Ca^2+^-activated force (Fₘₐₓ) in permeabilized rat and canine cardiomyocytes in a concentration-dependent manner at a sarcomere length (SL) of 2.3 µm (**A**, **B**). Approximate IC₅₀ values were 0.1 µM in rat and 0.2 µM in canine myocardium. Force measurements obtained at two SLs (1.9 µm, circles; 2.3 µm, squares) under drug-free (open symbols) and aficamten-treated conditions (closed symbols; 0.1 µM rat, 0.2 µM dog) enabled assessment of SL-dependent effects on active force (F_active_; **C**, **D**). Normalized pCa–force curves illustrate aficamten- and SL-dependent alterations in Ca^2+^ sensitivity (**E**, **F**). Data are presented as mean ± SEM from n = 3 rat and n = 3 canine hearts (6–8 cells total per species). Significant differences between aficamten-treated and control groups are indicated by asterisks (*P < 0.05). For clarity, individual significant changes are not shown in panels C–F

To further characterize the concentration-dependent effects of aficamten on myofilament function, permeabilized rat and canine LV cardiomyocytes were exposed to increasing concentrations of the drug (0.001–10 µM) under isometric conditions. In both species, maximal Ca^2+^-activated force (F_max_, measured at pCa 4.75) declined progressively with increasing aficamten concentrations, yielding IC₅₀ estimates of approximately 0.1 µM in rat and 0.2 µM in canine cardiomyocytes (Fig. 5A, B). To compare drug effects under graded Ca^2+^ activation, aficamten was applied at concentrations corresponding to the respective IC₅₀ values (0.1 µM for rat and 0.2 µM for dog), and pCa–force relationships were assessed between pCa 4.75 and 7.0 at two SLs: 1.9 µm and 2.3 µm. Drug-free measurements served as controls. In the absence of aficamten, stretching cardiomyocytes from 1.9 µm to 2.3 µm increased F_max_ from 8.43 ± 0.51 kN·m⁻^2^ to 15.73 ± 1.47 kN·m⁻^2^ in rats and from 11.40 ± 0.48 kN·m⁻^2^ to 17.62 ± 1.36 kN·m⁻^2^ in dogs, confirming the expected length-dependent activation. Aficamten produced comparable reductions in F_max_ in both species at both SLs. Specifically, F_max_ decreased to 5.04 ± 0.52 kN·m⁻^2^ at 1.9 µm and to 8.41 ± 1.31 kN·m⁻^2^ at 2.3 µm in rat cardiomyocytes, and to 6.67 ± 0.25 kN·m⁻^2^ at 1.9 µm and to 9.82 ± 0.66 kN·m⁻^2^ at 2.3 µm in canine cardiomyocytes (Fig. 5C, D). Normalization of submaximal Ca^2+^-activated force (F_active_) to F_max_ allowed analysis of Ca^2+^ sensitivity of force production (pCa₅₀). In drug-free conditions, increasing sarcomere length shifted pCa₅₀ to higher values in both species (from 5.75 ± 0.03 to 5.83 ± 0.05 in rats, P < 0.05; and from 5.80 ± 0.01 to 5.88 ± 0.03 in dogs, P < 0.05), consistent with enhanced Ca^2+^ sensitivity at longer sarcomere lengths. In contrast, aficamten abolished this length-dependent increase in Ca^2+^ sensitivity: pCa₅₀ values did not differ significantly between the two sarcomere lengths in either species (P > 0.05; Fig. 5E, F).

### Aficamten decreases passive stiffness of permeabilized rat and canine cardiomyocytes at high concentrations

**Fig. 6:**
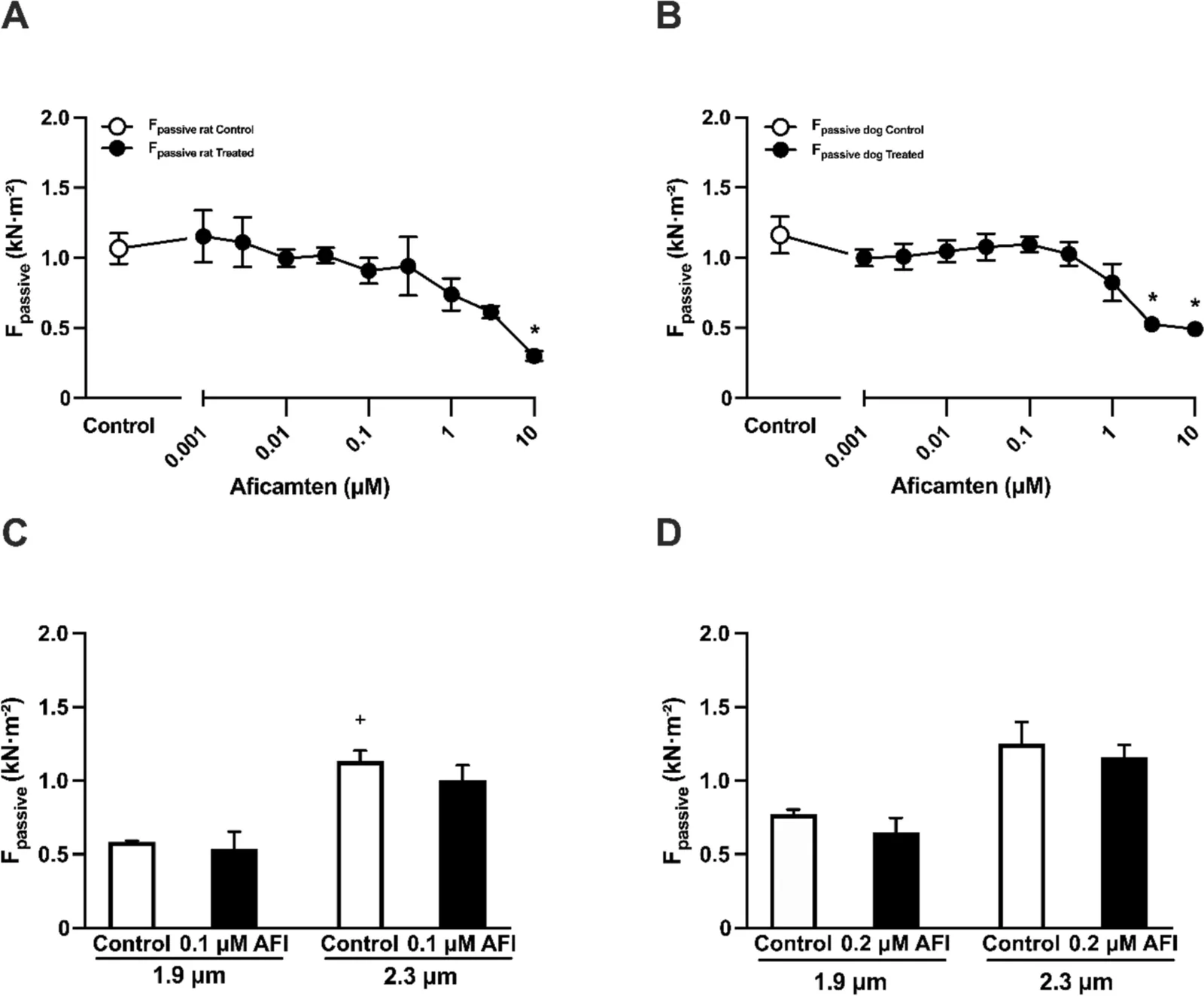
Concentration-dependent effects of aficamten on passive stiffness (F_passive_) in permeabilized single left ventricular rat (**A**, **C**) and canine (**B**, **D**) cardiomyocytes. Passive stiffness (F_passive_) was reduced only at high aficamten concentrations at an SL of 2.3 µm in both rat (**A**) and canine (**B**) cardiomyocytes. As expected, decreasing SL from 2.3 to 1.9 µm markedly reduced F_passive_; however, aficamten (0.1 µM rat; 0.2 µM dog) did not alter F_passive_ at either SL (**C**, **D**). Data are presented as mean ± SEM from n = 3 rat and n = 3 canine hearts (6–8 cells total per species). Significant differences between aficamten-treated and control (drug-free) conditions are indicated by asterisks (*P < 0.05). Significant reductions in F_passive_ resulting from decreasing SL from 2.3 µm to 1.9 µm are indicated by crosses (+ P < 0.05)

The effects of aficamten on passive stiffness (F_passive_) were examined in permeabilized rat and canine cardiomyocytes across a wide concentration range (0.001–10 µM). Aficamten reduced F_passive_ only at relatively high concentrations: significant decreases were observed at 3 µM and 10 µM in rat cardiomyocytes, and at 3 µM in canine cardiomyocytes at a sarcomere length of 2.3 µm (Fig. 6A, B). As expected, reducing sarcomere length from 2.3 to 1.9 µm lowered F_passive_. This length-dependent reduction was not altered by aficamten at concentrations corresponding to its apparent IC₅₀ values (0.1 µM in rats and 0.2 µM in canines; Fig. 6C, D), indicating that passive stiffness is unaffected at pharmacologically relevant doses.

## Discussion

The present study in healthy myocardium provides mechanistic insight into how the cardiac myosin inhibitor aficamten integrates into the multilevel regulation of myocardial performance. By combining in vivo, intact-cell, and skinned cardiomyocyte experiments across rat and canine preparations, we demonstrate that the aficamten-induced reduction in active force generation can be counter-regulated by a myofilament-specific mechanism related to the Frank–Starling response, thereby maintaining cardiac output through increased diastolic chamber volumes and preserved length-dependent force generation. These findings from healthy animal preparations extend current knowledge on myosin inhibition and highlight compensatory pathways that may be relevant for understanding hemodynamic responses during therapy in patients with HCM or HFpEF, pending validation in disease-relevant models.

We applied aficamten concentrations spanning a wider range (0.001–10 µM) than those used clinically (approximately 0.17–0.38 µM) [28, 50]. This approach amplified aficamten-specific myocardial responses and highlighted their relationship with experimental variables central to systolic and diastolic performance. In sedated rats, the observed increase in basal heart rate is plausibly explained by reflex β-adrenergic activation, leading to partial pacing capture at 240 bpm, similar to observations in feline HCM models treated with aficamten [44]. Changes in several cardiovascular parameters (e.g., LV dilation and substantial reductions in FS, EF, and GLS) were consistent with markedly depressed systolic performance in vivo. Nevertheless, SV and CO were maintained at 300 and 400 bpm—pacing frequencies well above the aficamten-induced baseline heart rate (233.6 bpm)—where external pacing overrides sympathetic compensation, providing controlled conditions for assessing true preload-mediated Frank-Starling compensation. Paired within-animal comparisons confirmed consistent EDV and ESV increases with preserved SV and CO across all animals at 300 bpm. Furthermore, aficamten increased the E/e' ratio and isovolumetric relaxation time (IVRT) at all pacing frequencies, indicating elevated left ventricular filling pressures consistent with preload recruitment and Frank-Starling compensation independent of neurohormonal activation.

In intact canine cardiomyocytes, reductions in systolic shortening, contraction duration, and time-to-peak shortening facilitated diastolic augmentation at different pacing rates. Notably, Ca^2+^ transient amplitudes and durations responded to increases in pacing frequency in the same manner in the absence and presence of aficamten. Together, these findings demonstrate that aficamten does not interfere with the intrinsic frequency-dependent acceleration of contraction and relaxation kinetics, nor with the physiological FFR and its coupled modulation of intracellular Ca^2+^ handling [34]. Instead, aficamten selectively reduces contraction amplitude while preserving frequency-driven Ca^2+^ dynamics. This indicates that its myosin-inhibitory action remains stable across a wide physiological range of heart rates and does not disrupt the fundamental rate-dependent properties of excitation–contraction coupling. Consistent with our findings, a recent study in intact canine cardiomyocytes demonstrated that aficamten reduces contraction amplitude without altering Ca^2+^ transients, a property considered therapeutically advantageous in arrhythmogenic substrates [32]. Notably, aficamten decreased contraction duration and, consequently, increased diastolic duration across stimulation frequencies, which may ameliorate the impaired lusitropic response characteristic of HCM and HFpEF, particularly under exercise or adrenergic stress [5, 21]. However, it should be noted that the concentration employed in the present study (0.4 µM) substantially reduces peak force and may exceed clinically relevant free myofilament exposures; consistent with this, a lower mavacamten concentration (0.33 µM) did not affect contraction duration in engineered heart tissue as previously reported [42], while 0.5 µM markedly shortened it, suggesting that kinetic effects are concentration-dependent and may be attenuated at therapeutic dosing.

Here we show that myofilament-generated active force decreased in a concentration-dependent manner with similar potency in rat and canine cardiomyocytes. Although length-dependent activation increased F_max_ and Ca^2+^ sensitivity under drug-free conditions, aficamten blunted the length-dependent enhancement of Ca^2+^ sensitivity while not limiting the increase in F_max_ with longer sarcomere lengths. These results indicate that aficamten exerts a consistent inhibitory action on cardiac myofilaments that only partially constrains physiological length-dependent regulatory mechanisms, thereby providing a mechanistic basis for the preservation of SV and CO despite its robust negative inotropic effects observed in vivo and in isolated cells.

Finally, we show that aficamten exerts minimal effects on passive cardiomyocyte stiffness at pharmacologically relevant concentrations. Thus, its mechanical action remains confined primarily to the modulation of active force generation, with the passive structural properties of the cardiomyocyte largely preserved.

The interaction between myosin inhibition and the Frank–Starling mechanism has been investigated primarily with mavacamten, which provides a relevant mechanistic framework for interpreting our findings with aficamten. In skinned human and porcine myocardium, mavacamten decreases F_max_ and Ca^2+^ sensitivity at a given sarcomere length, yet preserves the length-dependent increase in Ca^2+^ sensitivity, indicating that length-dependent activation remains largely intact at therapeutically relevant concentrations [2, 27]. Studies in engineered heart tissue similarly show a downward shift of the force–length relationship with retention of preload reserve under myosin inhibition [42]. Together, existing evidence suggests that despite reducing the pool of force-generating cross-bridges, mavacamten does not abolish length-dependent activation and allows normal Frank–Starling responsiveness to be maintained [2, 27, 42]. Our findings with aficamten extend and refine this framework. Aficamten produced a concentration-dependent suppression of myofilament force in both rat and canine cardiomyocytes, but importantly, preserved the length-dependent increase in Fₘₐₓ while attenuating the length-dependent increase in Ca^2+^ sensitivity. This partial dissociation between the Ca^2+^-sensitivity and maximal-force components of length-dependent activation suggests that, under aficamten, thick-filament–mediated recruitment of cross-bridges with stretch remains operative, whereas the thin-filament contribution to length-dependent activation becomes blunted. Such selective preservation of the thick-filament mechano-sensing pathway provides a mechanistic explanation for the maintained stroke volume and cardiac output observed in vivo, despite marked reductions in active force generation. Thus, our data position aficamten within a broader class-specific pattern—characterized by robust myosin inhibition with partial, but not complete, impairment of length-dependent activation—and highlight a compensatory sarcomere-level mechanism that supports Frank–Starling reserve during myosin-targeted therapy.

Hypercontractility and increased myofilament Ca^2+^ sensitivity are central to the pathophysiology of HCM, leading to increased energy demand, impaired relaxation, and mechano-energetic uncoupling [33, 40]. Neurohormonal activation, particularly via β-adrenergic stimulation, may further exacerbate hypercontractility and contribute to maladaptive remodeling [38]. Mutations that prolong sarcomere activation and delay relaxation [41, 46] can attenuate the Frank–Starling mechanism, limiting the ability of the ventricle to adjust force output across physiologic loading conditions [20]. In parallel, increased afterload due to septal hypertrophy and LVOT obstruction shifts the pressure–volume loop toward higher pressure and lower volume domains, reflecting reduced mechanical efficiency [32].

HFpEF is increasingly recognized as a disorder characterized not only by impaired diastolic relaxation and increased passive myocardial stiffness, but also by altered Frank–Starling reserve [22]. Multiple studies have demonstrated that patients with HFpEF exhibit a blunted length-dependent increase in stroke volume and contractility, reflecting impairments in both chamber-level preload responsiveness and underlying sarcomere length-dependent activation. Increased titin stiffness, extracellular matrix remodeling, and microvascular inflammation contribute to reduced diastolic filling reserve, while abnormalities in myofilament Ca^2+^ sensitivity and thick-filament regulation may limit the systolic component of the Frank–Starling response [3, 24, 25, 49, 52]. As a result, the ability of the HFpEF heart to augment stroke volume under physiological stress is substantially diminished, contributing to exercise intolerance and hemodynamic instability [4]. Understanding how pharmacologic interventions interact with this limited Frank–Starling reserve is therefore essential for therapeutic development in HFpEF as well.

Myosin inhibition has emerged as a rational therapeutic strategy targeting the fundamental abnormalities in HCM and HFpEF. Clinical and modeling studies with mavacamten demonstrated that reducing cross-bridge formation increases ventricular volumes, improves filling, and normalizes pressure–volume loops [32, 47]. Aficamten, a next-generation inhibitor, similarly reduces hypercontractility and improves functional capacity in patients with oHCM [7, 29]. The collective evidence suggests that increased diastolic chamber volumes—driven by augmented relaxation and increased ventricular compliance—enables ventricles treated with aficamten to operate at larger end-diastolic volumes. The recruitment of Frank–Starling reserve can thereby offset reductions in active force generation and maintain cardiac output even in the presence of negative inotropy. This compensation likely contributes to the favorable hemodynamic responses observed in clinical trials of aficamten, including improved exercise capacity and reduced LVOT gradients [7, 29]. The emerging clinical evidence that myosin inhibition can alleviate diastolic load and myocardial stress in HFpEF further underscores the relevance of our mechanistic observations, as the preserved length-dependent force reserve we identified under aficamten may represent a sarcomeric substrate through which CMIs could enhance diastolic filling and hemodynamic stability in appropriately selected HFpEF patients.

An important question raised by our findings is how aficamten's effects on length-dependent Ca^2+^ sensitivity may interact with protein kinase A (PKA)-mediated troponin I phosphorylation, a key regulator of myofilament Ca^2+^ sensitivity during adrenergic stress. In healthy myocardium, β-adrenergic stimulation promotes PKA-mediated phosphorylation of troponin I, which reduces Ca^2+^ sensitivity and accelerates relaxation [45]. However, in failing hearts, β-adrenergic receptor downregulation and altered phosphorylation patterns blunt this regulatory pathway [26]. In end-stage human heart failure, troponin I phosphorylation is markedly reduced, resulting in increased myofilament Ca^2+^ sensitivity [48], which may further limit the adaptive capacity of stress-activated signaling. Furthermore, length-dependent activation itself is often impaired in diseased myocardium, particularly in hypertrophic cardiomyopathy with sarcomeric mutations [41]. Our observation that aficamten abolishes length-dependent Ca^2+^ sensitization at IC_50_ concentrations raises the possibility that the drug may also modulate the phosphorylation-dependent component of Ca^2+^ sensitivity regulation. Importantly, clinical aficamten exposures are substantially lower (5–tenfold below IC_50_ when accounting for plasma protein binding), suggesting that both length-dependent and phosphorylation-dependent Ca^2+^ sensitivity modulation may be partially preserved at therapeutic doses [6]. Nevertheless, future studies examining aficamten's effects in the presence of PKA stimulation, in diseased myocardium with altered baseline phosphorylation states, and across a range of clinically-relevant aficamten and Ca^2+^ concentrations will be essential to fully understand how myosin inhibition integrates with stress-activated signaling pathways.

Limitations of the present study should be acknowledged. Most notably, our experiments were conducted in cardiomyocytes and myocardial preparations derived from healthy rat and canine hearts. Although this approach allowed precise characterization of myofilament-level mechanisms, studies in healthy myocardium may not fully capture disease-specific interactions—including phosphorylation-dependent modulation of Ca^2+^ sensitivity—that may be relevant in failing or hypertrophic hearts with altered baseline phosphorylation patterns and blunted β-adrenergic responsiveness [16]. Because cardiac myosin inhibitor therapy is primarily intended for patients with HCM or HFpEF, disease-specific alterations in sarcomere structure, passive stiffness, Ca^2+^ handling, and thick-filament regulation [35] may modulate the quantitative effects of aficamten on force generation and length-dependent activation. Nevertheless, the use of two phylogenetically distinct species—with rat myocardium dominated by α-myosin heavy chain (MHC) and canine myocardium dominated by β-MHC—allowed us to assess sarcomere-level drug effects across different myosin isoform compositions [8, 11]. The high degree of concordance between the two models (despite additional differences in their FFRs) supports the robustness and generalizability of the mechanistic principles we observed. Moreover, the integration of in vivo, intact-cell, and skinned cardiomyocyte measurements provides internal cross-validation and reduces the likelihood that the observed effects reflect species- or preparation-specific artifacts. Additionally, intact cardiomyocyte experiments were conducted at room temperature and low pacing rates, which are standard conditions for cross-species comparisons but may not fully recapitulate physiological excitation–contraction coupling dynamics. Future studies in genetically defined HCM models, HFpEF myocardium, or human engineered cardiac tissues will be essential to determine how disease-specific remodeling interacts with aficamten’s myofilament actions.

Taken together, our findings in healthy myocardium provide a mechanistic framework explaining how myosin inhibition can reduce contractility while maintaining cardiac output. By simultaneously increasing diastolic chamber volumes and preserving length-dependent force generation, aficamten leverages intrinsic cardiac compensatory mechanisms that may contribute to hemodynamic stability in appropriately selected patients, offering a physiological rationale for its continued therapeutic development in HCM and HFpEF [15].

## Supplementary Information

Below is the link to the electronic supplementary material.

Supplementary file1 (TIF 778 KB)

Supplementary file2 (TIFF 1990 KB)

Supplementary file3 (TIFF 1277 KB)

## Data Availability

The datasets generated during the current study are available from the corresponding author on reasonable request.
